# Predictive models of severe disease in patients with COVID-19 pneumonia at an early stage on CT images using topological properties

**DOI:** 10.1007/s12194-025-00906-1

**Published:** 2025-04-28

**Authors:** Takahiro Iwasaki, Hidetaka Arimura, Shohei Inui, Takumi Kodama, Yun Hao Cui, Kenta Ninomiya, Hideyuki Iwanaga, Toshihiro Hayashi, Osamu Abe

**Affiliations:** 1 Division of Medical Quantum Science, Department of Health Sciences, Graduate School of Medical Sciences, Kyushu University, Fukuoka, Japan; 2 Department of Radiology, The University of Tokyo Hospital, Tokyo, Japan; 3 Division of Medical Quantum Science, Department of Health Sciences, Faculty of Medical Sciences, Kyushu University, Fukuoka, Japan; 4 Department of Radiology, Graduate School of Medicine, The University of Tokyo, Tokyo, Japan; 5 Harry Perkins Institute of Medical Research, The University of Western Australia, Western Australia, Australia; 6 Division of Financial Strategy Management, The University of Tokyo Hospital, Tokyo, Japan

**Keywords:** COVID-19, Severity, Topological features, Accumulated Betti number map, Predictive model

## Abstract

Prediction of severe disease (SVD) in patients with coronavirus disease (COVID-19) pneumonia at an early stage could allow for more appropriate triage and improve patient prognosis. Moreover, the visualization of the topological properties of COVID-19 pneumonia could help clinical physicians describe the reasons for their decisions. We aimed to construct predictive models of SVD in patients with COVID-19 pneumonia at an early stage on computed tomography (CT) images using SVD-specific features that can be visualized on accumulated Betti number (BN) maps. BN maps (b0 and b1 maps) were generated by calculating the BNs within a shifting kernel in a manner similar to a convolution. Accumulated BN maps were constructed by summing BN maps (b0 and b1 maps) derived from a range of multiple-threshold values. Topological features were computed as intrinsic topological properties of COVID-19 pneumonia from the accumulated BN maps. Predictive models of SVD were constructed with two feature selection methods and three machine learning models using nested fivefold cross-validation. The proposed model achieved an area under the receiver-operating characteristic curve of 0.854 and a sensitivity of 0.908 in a test fold. These results suggested that topological image features could characterize COVID-19 pneumonia at an early stage as SVD.

**Supplementary Information:**

The online version contains supplementary material available at 10.1007/s12194-025-00906-1.

## Introduction

The coronavirus disease 2019 (COVID-19) pandemic has spread worldwide from 2019 to May 2023 [1]. According to the World Health Organization (WHO), 777,594,331 cases had been confirmed as COVID-19 and 7,089,989 cases had died as of March 2025 [2]. COVID-19 is caused by the severe acute respiratory syndrome coronavirus 2 (SARS-CoV-2), which causes fever, persistent cough, and shortness of breath [3]. According to the National Health Commission of the People’s Republic of China, the severity of COVID-19 can be grouped into moderate, mild, severe, and critically ill based on symptoms and clinical examination [4]. Patients in critically ill and severe forms are described as severe disease (SVD) and patients in regular and mild forms as non-severe disease (non-SVD) [5, 6]. SVD patients experience respiratory failure due to low oxygen saturation (≤ 93%) [4]. Although the WHO declared the end of the public health emergency of international concern (PHEIC) state caused by COVID-19 in May 2023 [1], patients with COVID-19 have still suffered worldwide and the number of cases has also increased (13,183,604 new cases and 162,137 deaths after the end of PHEIC) [2]. Therefore, prediction of SVD in patients with COVID-19 pneumonia at an early stage could allow for more appropriate triage and improve their prognosis.

In the diagnosis of COVID-19, computed tomography (CT) is used to assess lung regions and plays a leading role as one of the clinical evidences alongside reverse transcription-polymerase chain reaction (RT-PCR). COVID-19 pneumonia presents various CT findings, including ground-glass opacity (GGO), consolidation, crazy-paving sign, sieve-hole sign, and cavity sign depending on the severity [7–10]. However, the evaluation of these findings depends on radiologists and is subjective [6]. We noted that these findings, such as GGO or consolidation, consist of topological structures such as lumps and holes from a mathematical point of view. The topology derived from the lumps and holes may detect the potential signs of progression to SVD in the future.

Topological properties can be characterized using Betti number (BN), which is mathematically invariant. The Betti number in a two-dimensional (2D) space consists of 0D BN (b0) and 1D BN (b1), which represent the numbers of lumps (or connected components) and holes, respectively [11]. Ninomiya et al. reported that topological features of BN maps, whose pixels represent BN within a specific kernel, could predict symptomatic (grade 2) radiation-induced pneumonia before stereotactic ablative radiotherapy for lung cancer [12]. Therefore, we assumed that the BN maps could characterize COVID-19 pneumonia as well.

Previous studies have reported the feasibility of radiomic features [6, 13, 14] and deep learning [15–18] for COVID-19 pneumonia for prediction or detection [19, 20] of SVD on CT images. In particular, the gradient-weighted class activation mapping (Grad-CAM), which indicates heatmaps to visualize areas with regions of interest on images, is expected to improve the accountability of artificial intelligence [21–24]. However, Grad-CAM is generated from convolutional neural networks (CNNs), which could not be interpretable. In addition, Grad-CAM is a low-resolution and high-value area spread with a slow gradient in heat maps. In contrast, since BN maps could reflect topological properties of COVID-19 pneumonia, the outputs of predictive models would be interpretable with higher resolutions in heat maps than conventional methods. Visualization of the topological properties of COVID-19 pneumonia could help clinical physicians describe the reasons for their decisions.

We hypothesized that the topological properties from the accumulated BN maps could be informative for the prediction of SVD in patients with COVID-19 pneumonia at an early stage. The aim of this study was to construct predictive models of SVD in patients with COVID-19 pneumonia at an early stage on CT images using SVD-specific features that can be visualized on accumulated Betti number (BN) maps.

## Materials and methods

### Patient information

Table 1 summarizes the patient information used in this study. The CT images of 258 patients with COVID-19, consisting of 94 SVD and 164 non-SVD patients, were collected from an open database provided by Huazhong University of Science and Technology [18]. All patients underwent RT-PCR and CT scanning at the Union Hospital (China) and the Liyuan Hospital (China). The pneumonia regions were segmented using Otsu’s automatic thresholding technique [25], because it is difficult to determine the precise pneumonia region in clinical situations.

**Table 1:** Patient information used in this study

		Severe disease (SVD) (*n* = 94)		Non-SVD (*n* = 164)	*p* value
		Critically ill	Severe	Regular	
No. of cases		12	82	164	–
Sex	Male	7	39	86	0.588 (Chi-squared test)
	Female	5	43	78	
Age	Median	72.5	61	58	3.56 × 10^–3^ (Mann–Whitney *U* test)
	Range	46–93	32–91	9–92	

Table 2 shows the patient information of 60 cases with annotated pneumonia regions for the first and second feature selections. Sixty patients, consisting of 30 SVD and 30 non-SVD patients, were randomly selected from the 258 patients. The pneumonia regions were segmented using Otsu’s automatic thresholding technique [25] and manually corrected using a 3D slicer [26]. A radiologist (S.I.) validated the pneumonia regions. The 60 patients were included in the training data.

**Table 2:** Patient information of 60 cases with annotated pneumonia regions for the first and second feature selections

		Severe disease (SVD) (*n* = 30)		Non-SVD (*n* = 30)	*p* value
		Critically ill	Severe	Regular	
No. of cases		6	24	30	–
Sex	Male	3	12	13	0.608 (Chi-squared test)
	Female	3	12	17	
Age	Median	76.5	63	59	0.153 (Mann–Whitney *U* test)
	Range	46–93	32–85	17–88	

The patients were scanned on various CT scanners [TOSHIBA (Japan), SIEMENS (Germany), PHILIPS (Netherlands), and United Imaging Healthcare (China)] with voltages of 100 or 120 kV, pixel sizes of 0.633 mm^2^ to 0.977 mm^2^, slice thicknesses of 1.5 mm–2.0 mm, and a matrix size of 512 × 512. A single slice containing the pneumonia region was selected for each patient.

### Overall workflow

**Fig. 1:**
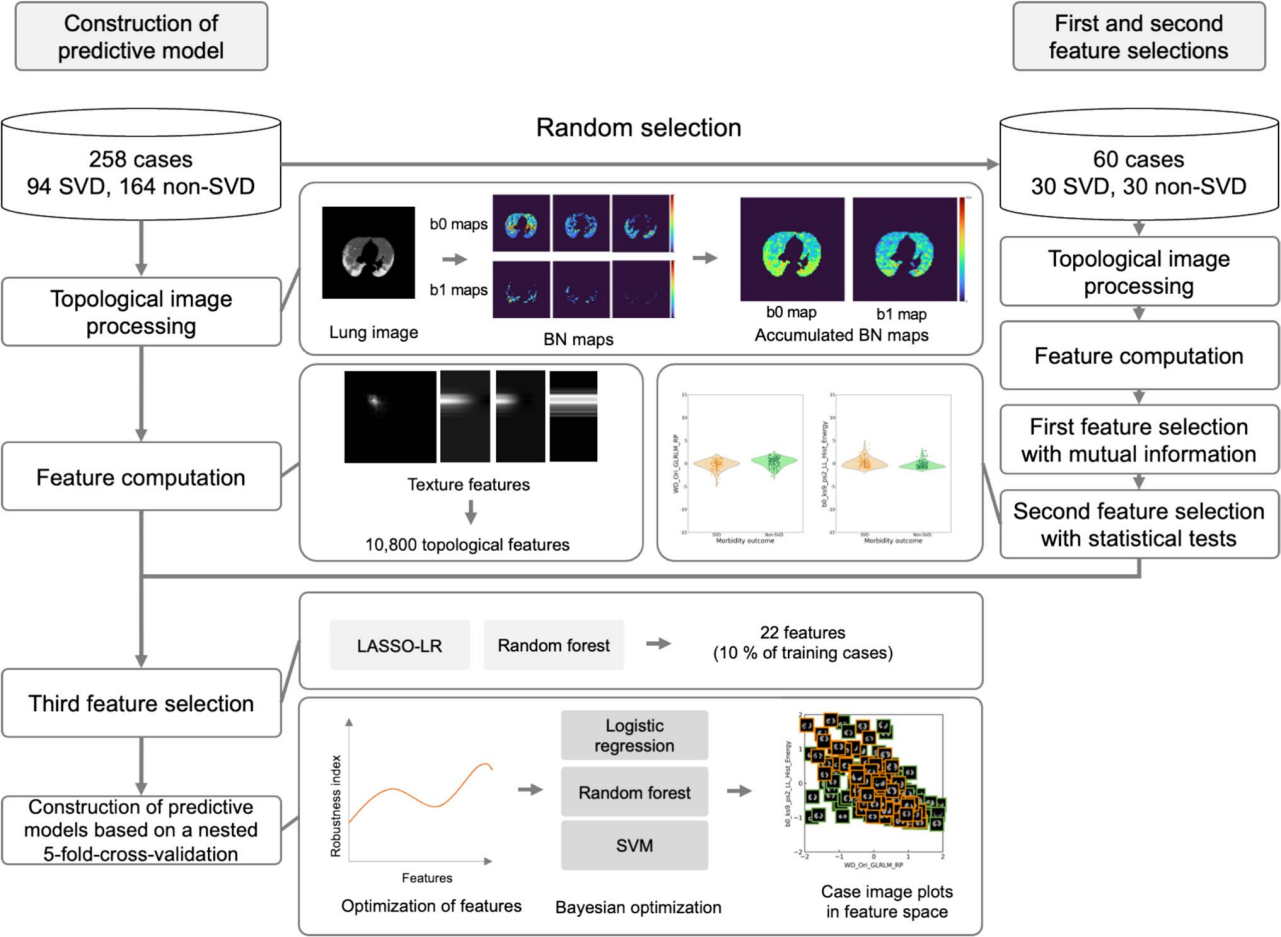
Overall workflow for constructing a predictive model of SVD in patients with COVID-19 pneumonia using accumulated BN maps

Figure 1 shows the overall workflow for constructing a predictive model of SVD in patients with COVID-19 pneumonia using accumulated BN maps. A total of 258 patients were randomly divided into 198 patients for model construction and 60 patients for first and second feature selections. BN maps were generated in topological image processing by calculating BNs (b0 and b1) within a shifting kernel in a manner similar to a convolution. BN maps derived from a range of threshold values were accumulated for each BN map for topological feature computation. In the first and second feature selections, significant feature candidates were selected from the features computed in the pneumonia regions of 60 cases based on the p values of statistical tests following mutual information between SVD and non-SVD patients. Subsequently, two feature selection methods were deployed for the third feature selection. Predictive models of SVD were constructed using nested fivefold cross-validation (CV) encompassing the training and test datasets, as shown in Supplementary Fig. 1 (Online Resource 1). The nested fivefold CV was leveraged to estimate unbiased results and reduce the chance of information leakage which could make our models stable and reproducible [27–29]. In the fivefold nested CV, the numbers of training and test cases were 208 or 206 (76 or 75 SVD, 131 or 132 non-SVD) and 50 or 52 (18 or 19 SVD, 32 or 33 non-SVD), respectively, depending on the fold.

### Topological image processing

94 SVD patients and 164 non-SVD patients were used to construct accumulated BN maps within lung regions. The lung regions were segmented using Otsu’s automatic thresholding technique [25]. Lung images were obtained by cropping the lung regions from the original CT images. The lung images were transformed into isotropic images with a pixel size of 0.98 × 0.98 mm^2^ using cubic and shape-based interpolations [30], during which the gray-scale values from -1350 to 150 Hounsfield units were normalized to 0 to 255 scale to suppress pixel value variations.

**Fig. 2:**
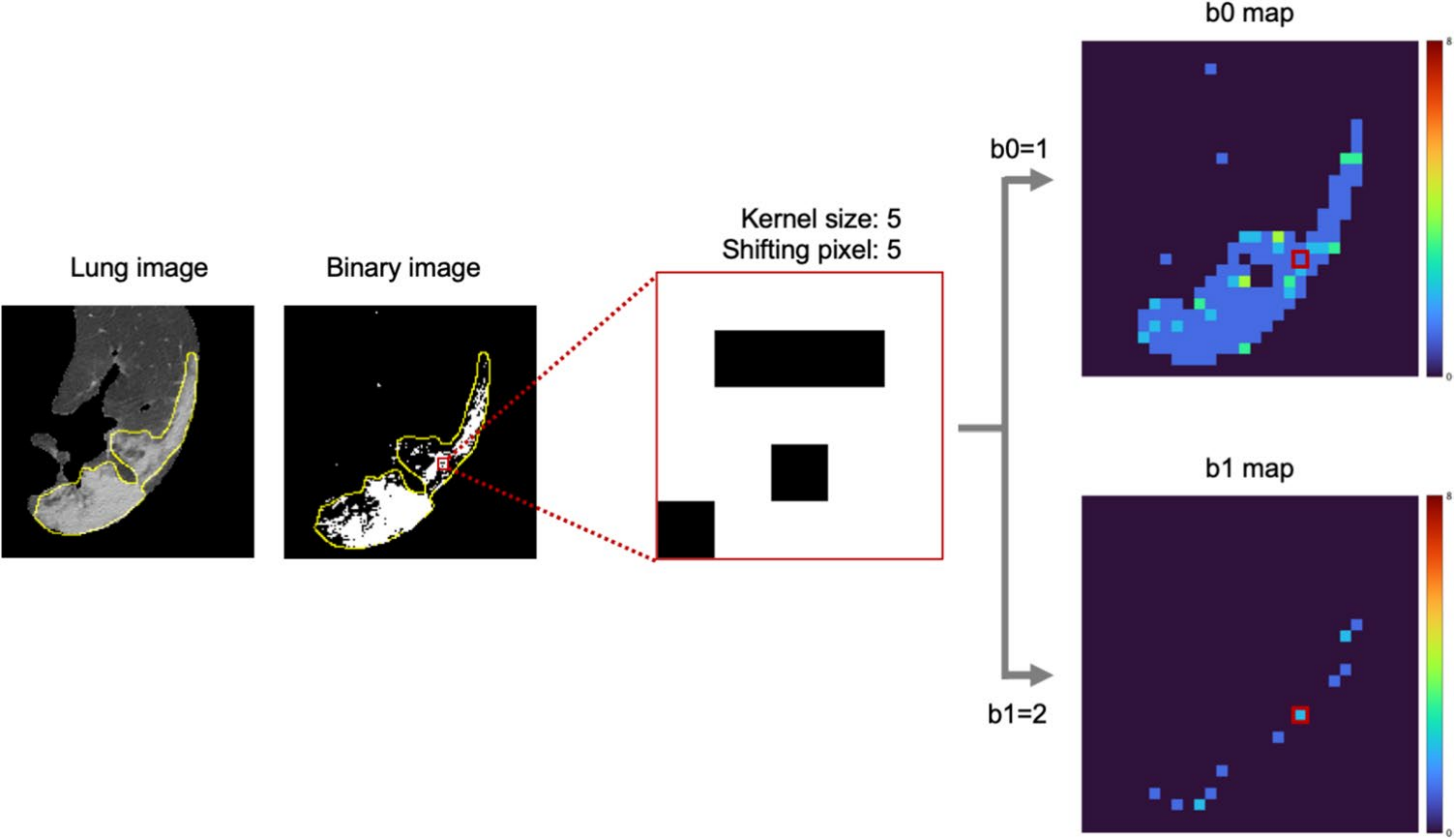
Calculation of b0 and b1 maps in a binary image obtained from a lung image. A pneumonia region is shown by yellow line. The kernel with a size of 5 pixels and shifting pixel of 5 were utilized for this case. In enlarged area (enclosed by red line), white areas represent the connected components and black areas represent the hole. Since b0 represents the number of connected components (white area), the number of connected components is one, which means b0 = 1. b1 represents the number of holes (black area) and there are two black area enclosed by white area, which means b1 = 2. Color bars indicate the Betti number from dark blue to dark red corresponding to 0 to higher numbers

Following the preprocessing, the lung images were consecutively binarized by multiple-threshold values to calculate the corresponding BN maps, followed by the accumulation of BN maps. Figure 2 illustrates the calculation of b0 and b1 maps in a binary image obtained from a lung image. The b0 and b1 were computed within a square kernel, and then, those values were placed at a pixel corresponding to the center pixel of the kernel in a manner similar to a mathematical convolution. The resultant images of b0 and b1 were referred to as b0 and b1 maps, respectively, which indicate the topological properties as color maps. The b0 and b1 maps represent maps of the numbers of connected and hole components, respectively.

**Fig. 3:**
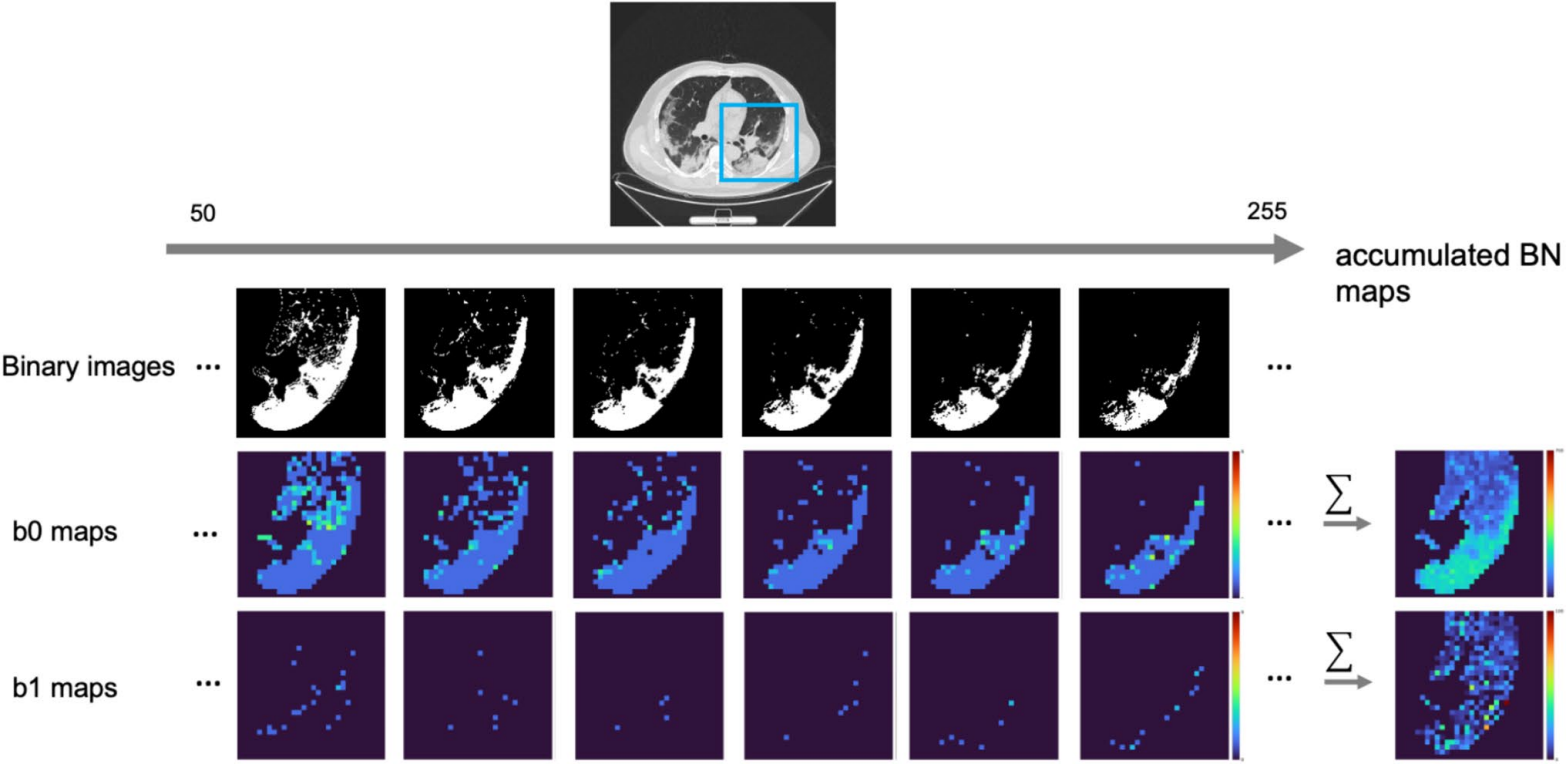
An original image, binary images consecutively binarized by multiple-threshold values from 50 to 255 with increments of 1, and their corresponding b0 and b1 maps. The accumulated Betti number (BN) maps are shown in the last column. The kernel with a size of 5 and shifting pixel of 5 were utilized for this case. Color bars indicate the BNs or accumulated BNs from dark blue to dark red corresponding to 0 to higher numbers

Figure 3 shows an original image, binary images consecutively binarized by multiple-threshold values from 50 to 255 with increments of 1, and their corresponding b0 and b1 maps. The accumulated BN maps are shown in the last column. The method for determining the initial threshold value for accumulated BN maps is described in Online Resource 2. For each binary image, b0 and b1 were computed within a square kernel, as shown in Fig. 2.

All b0 and b1 maps generated with the same kernel size and shifting pixel were summed to construct the accumulated b0 and b1 maps over consecutive threshold values from 50 to 255, as shown in Fig. 3. The kernel sizes of 5, 7, 9, and 11 pixels and shifting pixels of 1, 2, 3, 4, and 5 pixels were applied to the construction of the accumulated BN maps [12].

### Feature computation

A total of 10,800 topological features were obtained for each patient from 270 radiomic features × 2 accumulated BN maps (b0 and b1) × 4 kernel sizes × 5 shifting pixels. The radiomic features were composed of 14 histogram and 40 texture features in 5 types of images [an original accumulated BN map and four wavelet-decomposed (WD) images]. Gray-level co-occurrence matrix (GLCM), gray-level run-length matrix (GLRLM), gray-level size zone matrix (GLSZM), and neighborhood gray-tone difference matrix (NGTDM) were used to extract texture features. Wavelet decomposition was applied to the accumulated BN maps to obtain four WD images using the high (H)-pass filter and low (L)-pass filter based on the coiflet1 mother function [LL, LH, HL, and HH (each of the characters, L or H, represents a low-pass or high-pass filter, respectively)]. The topological features were corrected using harmonization [31–33]. In addition, topological features were augmented using the Synthetic Minority Over-sampling Technique (SMOTE) to improve the stability of predictive models with a small dataset. These features were then standardized to z-scores by the training dataset in every outer loop of the nested fivefold CV [34]. The feature computation was performed using a MATLAB-based radiomics tool package (MATLAB 2024a, MathWorks) [35].

### Feature selection

**Fig. 4:**
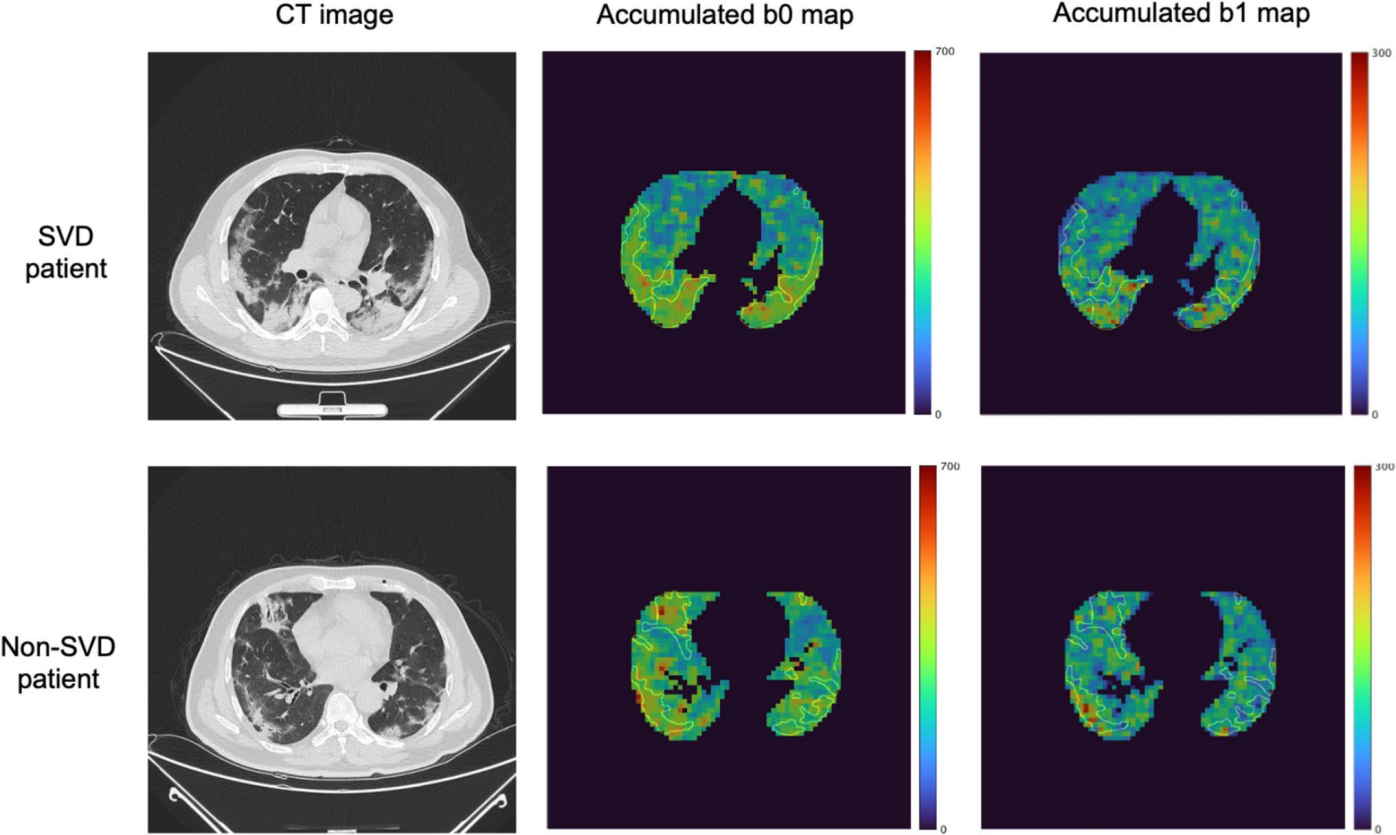
Pneumonia regions on original CT images for an SVD patient and a non-SVD patient and their corresponding accumulated BN maps with contours of the pneumonia regions. Color bars indicate the accumulated BNs from dark blue to dark red corresponding to 0 to higher numbers

The significant features were selected in three steps. In the first feature selection, significant feature candidates were selected from the features computed in the pneumonia regions of 60 cases (Table 2) based on mutual information between SVD and non-SVD patients. Figure 4 depicts the pneumonia regions on the original CT images for an SVD patient and a non-SVD patient and their corresponding accumulated BN maps with contours of the pneumonia regions. After computing the topological features of 30 SVD patients and 30 non-SVD patients, the number of features was decreased using mutual information to 108 (1.0% of 10,800) [36]. In the second feature selection, statistical tests between SVD and non-SVD patients were performed. The significance level was set at *p* < 0.05. The normality of the features was validated using Shapiro–Wilk test. When p values were lower than 0.05, Mann–Whitney’s *U* tests were applied to the features. When p values were 0.05 or more, an analysis of variance was performed. Welch’s *t* tests were applied to the features with p values lower than 0.05, whereas Student’s *t* tests were applied to the other features. The *p* values were corrected using the false discovery rate (FDR) to account for multiple comparisons [37].

Two feature selection methods, absolute values of coefficients of logistic regression with least absolute shrinkage and selection operator (LASSO-LR) or feature importance obtained from random forest, were deployed for the third feature selection. The significant features were selected in every outer loop of the nested fivefold CV. The number of significant features was limited to 5–10% of the number of training cases to reduce the overfitting problem [38–40].

### Construction of predictive models

Predictive models with Bayesian optimization were constructed using three predictive models following feature optimization [41]. In feature optimization, the predictive models were constructed by changing the number of features from 2 to 22 with fixed parameters and fivefold CV following the features selection. As predictive models, logistic regression (LR) [42], random forest (RF) [43], or support vector machine (SVM) [44] were applied. The number of features that achieved the highest robustness index (RI) was determined as the optimized number of features [45]. RI was defined as

$$\text{RI}=\frac{{\text{AUC}}_{\text{v}}}{1+\left|{\text{AUC}}_{\text{t}} - {\text{AUC}}_{\text{v}}\right|},$$

where ${\text{AUC}}_{\text{t}}$ and ${\text{AUC}}_{\text{v}}$ indicate the area under the receiver-operating characteristic curves (AUCs) for the identification of SVD patients in the training and validation procedures, respectively.

Following feature optimization, predictive models were constructed using Bayesian optimization [41]. Predictive models were constructed using the same models (LR, RF, and SVM) as those used for the feature optimization. The ranges for tuning the hyper-parameters are listed in Supplementary Table 1 (Online Resource 3). The predictive model with the highest RI was determined as the final model. These models were evaluated for sensitivities, specificities, accuracies, and AUCs for the test datasets. A predictive model using wavelet-decomposed (WD) images, which were generated from the lung image, was also constructed as conventional model.

Two combined feature sets, all-combined features and half-combined features, were also created using both topological and conventional features. The all-combined features were defined as a set of all features (topological and conventional features), which should be limited to 22 features. The half-combined features were defined as a set of 11 topological and 11 conventional features. The predictive model was constructed using “scikit-learn” package (version 1.3.2) on Python 3.9 and R (version 4.4.0) [46].

Case image plots in feature spaces were generated by plotting images instead of data using a combination of features that could achieve the best predictive model. The top three features were selected, which were used in constructing the best predictive model and showed statistically significant differences in both the validation and test data. The case image plots were visualized with the highest accuracy for the classification of SVD and non-SVD patients in the validation dataset using quadratic discriminant analysis (QDA).

## Results

**Fig. 5:**
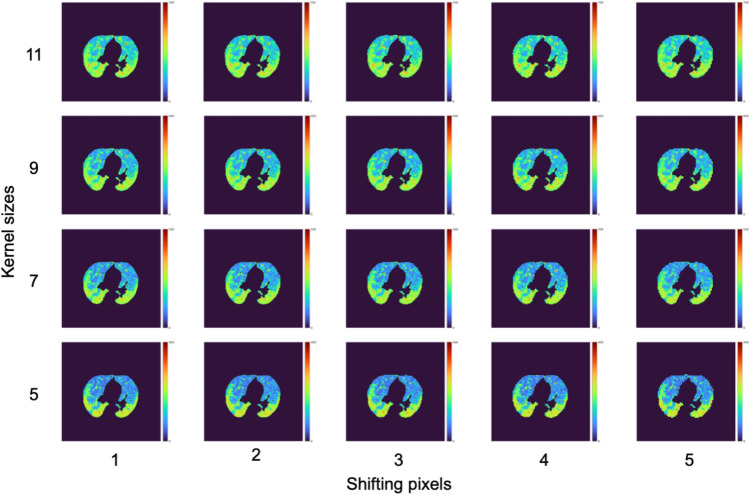
Accumulated b0 maps of an SVD patient for different kernel sizes and shifting pixels. Color bars indicate the accumulated BNs from dark blue to dark red corresponding to 0 to higher numbers

**Fig. 6:**
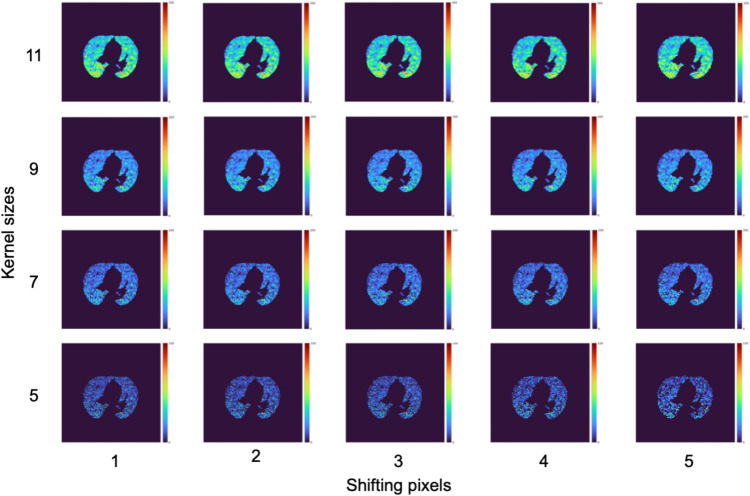
Accumulated b1 maps of an SVD patient for different kernel sizes and shifting pixels. Color bars indicate the accumulated BNs from dark blue to dark red corresponding to 0 to higher numbers

Topological properties of COVID-19 pneumonia were visualized as shown in Figs. 5 and 6. A total of 40 accumulated BN maps were constructed by combining four kernel sizes and five shifting pixels for the b0 and b1 maps.

Table 3 shows the mean sensitivities, specificities, accuracies, and AUCs for the validation and test in the nested fivefold CV. A predictive model using topological features achieved a sensitivity of 0.525, a specificity of 0.689, an accuracy of 0.637, and an AUC of 0.643 in the test using RF with 12–22 features selected by RF. In contrast, a predictive model using conventional features achieved a sensitivity of 0.575, a specificity of 0.701, an accuracy of 0.662, and an AUC of 0.660 in the test using LR with 15–21 features selected by LASSO-LR. For combined features, a predictive model using half-combined features achieved a lower AUC of 0.613 in the test using RF with 21–22 features selected by RF. A predictive model using all-combined features could achieve a higher AUC of 0.692 in the test using RF with 14–22 features selected by LASSO-LR. The detailed scores of the outer loops and the mean scores for the nested fivefold CV are in Supplementary Tables 2 and 3 (Online Resources 4 and 5).

**Table 3:** Mean sensitivities, specificities, accuracies, and areas under the characteristic curves (AUCs) for validation and test in a nested fivefold cross-validation

Features	Correction	No. of features	Feature selection	Machine learning		Sensitivity	Specificity	Accuracy	AUC
Topological features	Harmonization	12–22	RF	RF	Validation	0.801	0.738	0.769	0.860
						(0.774–0.826)	(0.722–0.760)	(0.752–0.778)	(0.852–0.870)
					Test	0.525	0.689	0.637	0.643
						(0.415–0.662)	(0.622–0.741)	(0.555–0.685)	(0.516–0.718)
Conventional features	Original	15–21	LASSO-LR	LR	Validation	0.776	0.743	0.759	0.836
						(0.731–0.796)	(0.708–0.781)	(0.730–0.788)	(0.820–0.861)
					Test	0.575	0.701	0.662	0.660
						(0.383–0.923)	(0.369–0.852)	(0.554–0.730)	(0.580–0.744)
Half-combined features	Harmonization	21–22	RF	RF	Validation	0.792	0.738	0.765	0.846
						(0.753–0.832)	(0.724–0.746)	(0.741–0.778)	(0.823–0.867)
					Test	0.497	0.667	0.612	0.613
						(0.354–0.569)	(0.631–0.726)	(0.560–0.665)	(0.513–0.680)
All-combined features	Original	14–22	LASSO-LR	RF	Validation	0.775	0.772	0.774	0.854
						(0.723–0.816)	(0.745–0.795)	(0.748–0.799)	(0.844–0.863)
					Test	0.579	0.714	0.672	0.692
						(0.450–0.908)	(0.654–0.807)	(0.605–0.738)	(0.569–0.854)

*LASSO* logistic regression with least absolute shrinkage and selection operator, *LR* logistic regression, *SVM* support vector machine, *RF* random forest

Table 4 shows the comparisons of sensitivities, specificities, accuracies and AUCs in the test between the best model in this study and previous studies. The best predictive model using the conventional features achieved a sensitivity of 0.600, a specificity of 0.793, and an AUC of 0.744 in the test using LR with 16 features selected by LASSO-LR. In contrast, the best predictive model using all-combined features achieved a sensitivity of 0.908, a specificity of 0.654, and an AUC of 0.854 in the test using RF with 22 all-combined features selected by LASSO-LR. We discuss the comparisons with previous studies in Discussion section. The selected features of all-combined features for the best predictive model are listed in Supplementary Table 4 (Online Resource 6).

**Table 4:** Comparisons of sensitivities, specificities, accuracies, and areas under the characteristic curves (AUCs) in the test between the best model in this study and previous studies

Study	No. of cases (training:test)	Internal validation	Features (no. of features)	Feature selection Machine learning	Sensitivity	Specificity	AUC
This study (All-combined features)	258 (208:50)	Nested fivefold CV	Original topological features and radiomic features (No. of features: 22)	LASSO-LR RF	0.908	0.654	0.854
Conventional features	258 (208:50)	Nested fivefold CV	Original radiomic features (No. of features: 16)	LASSO-LR LR	0.600	0.793	0.744
Xie et al., 2021	150 (105:45)	tenfold CV	Radiomic features	mRMR and LASSO Multivariate regression	0.650	0.990	0.980
Tang et al., 2021	118 (79:39)	threefold CV	Quantitative features Radiomic features	RF RF	0.900	0.830	0.980
Xiao et al., 2020	408 (303:105)	fivefold CV	Deep learning	- ResNet-34	0.875	0.785	0.892
Chieregato et al., 2022	558 (451:107)	tenfold CV	Deep learning Clinical data	Boruta CatBoost	0.839	0.934	0.949

*LASSO* logistic regression with least absolute shrinkage and selection operator, *LR* logistic regression, *RF* random forest, *mRMR* max-relevance and min-redundancy, *CV* cross validation

**Fig. 7:**
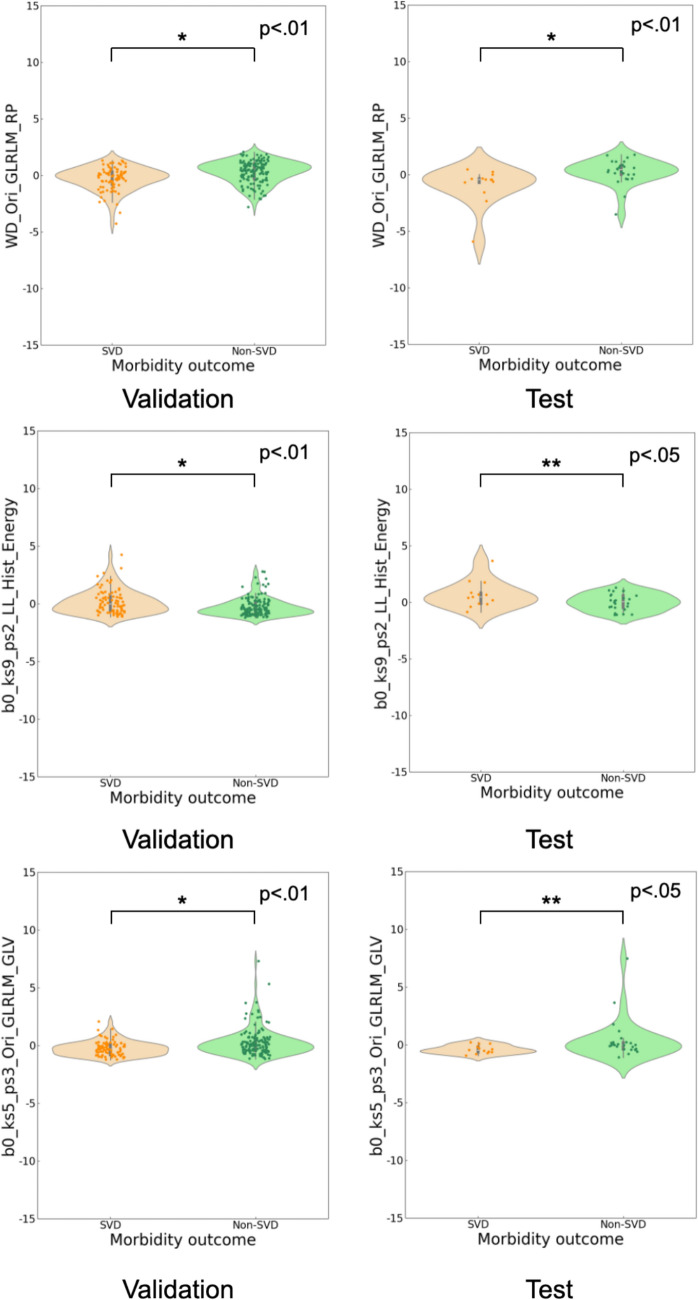
Violin plots of the top three features used in constructing the best predictive model, which showed statistically significant differences in both the validation and test data

Figure 7 shows violin plots of the top three features used in constructing the best predictive model, which showed statistically significant differences in both the validation and test. These features were composed of WD_Ori_GLRLM_RP, b0_ks9_ps2_LL_Hist_Energy, and b0_ks5_ps3_Ori_GLRLM_GLV.

**Fig. 8:**
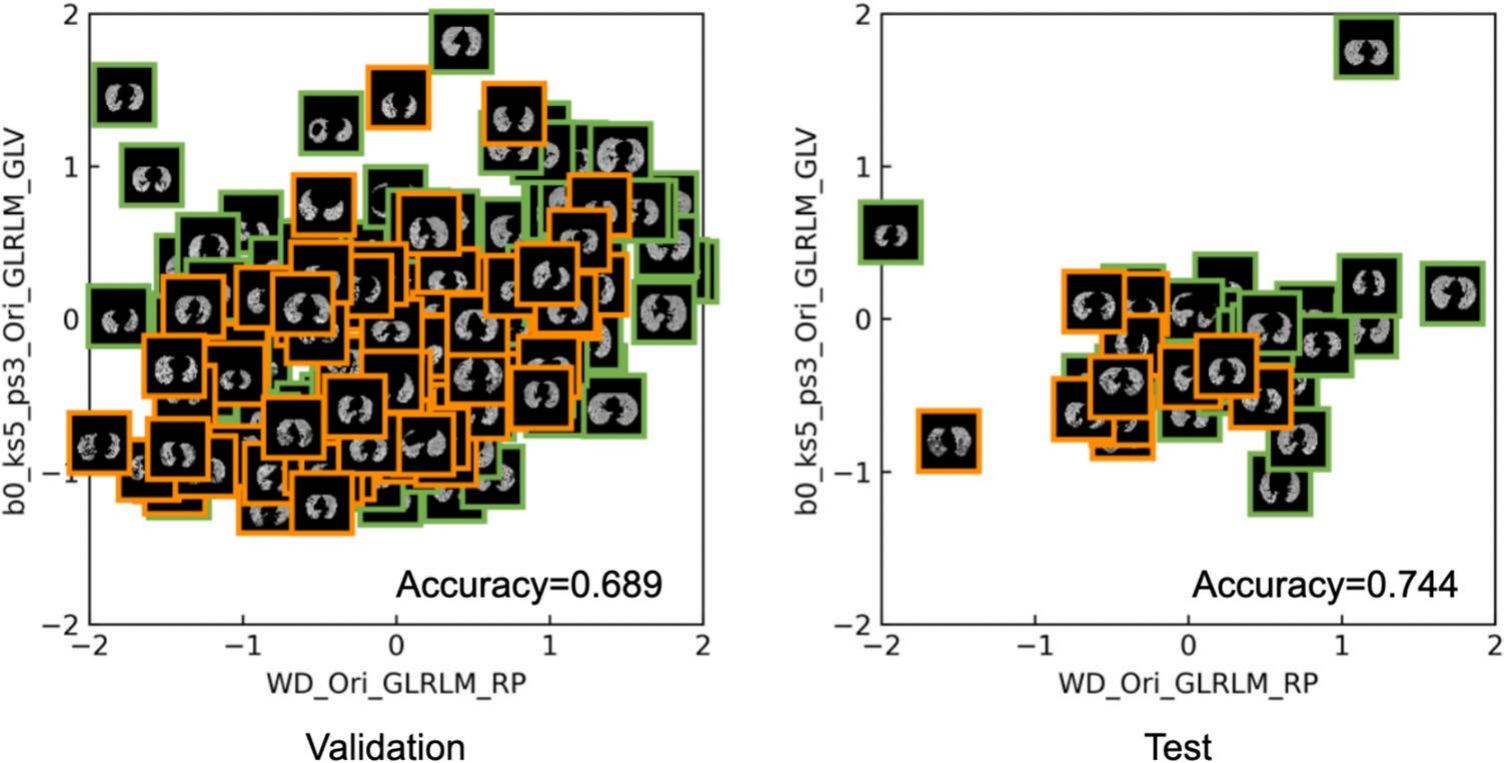
Case image plots in feature space from the selected features that contributed the best predictive model of SVD patients (orange squares) and non-SVD patients (green squares). The plot that achieved the best accuracy (0.689) in the validation was selected

In addition, Fig. 8 shows the case image plots in feature space from the selected features that contributed the best predictive model of SVD. The best accuracy achieved by QDA was 0.689 in the validation and 0.744 in the test with a combination of WD_Ori_GLRLM_RP and b0_ks5_ps3_Ori_GLRLM_GLV. The accuracies of the QDA with combinations of the three features are summarized in Supplementary Table 5 (Online Resource 7).

## Discussion

In this study, a predictive model of SVD in patients with COVID-19 pneumonia was constructed following the construction of accumulated BN maps. The predictive model of SVD using both topological and conventional features achieved an AUC of 0.854 and a sensitivity of 0.908 in a test fold.

Twenty accumulated b0 maps and 20 accumulated b1 maps were constructed for each patient. The accumulated b0 maps (Fig. 5) reflected the lumps in the images. Visually, the pneumonia regions could be visualized on the accumulated b0 maps with a small kernel size. The information of the holes was also reflected in the accumulated b1 maps (Fig. 6). There was no coincidence in the pneumonia region, whereas some higher-value spots could be visualized in the pneumonia regions. Accumulated BN maps were constructed prior to constructing predictive models. Therefore, topological properties of COVID-19 pneumonia could be considered in contrast to Grad-CAM. In addition, an original BN map needs to determine an appropriate threshold value for generation. The accumulation of BN maps could avoid fine-tuning of the threshold values.

Predictive models of SVD patients were constructed using topological and conventional features. Independently, a predictive model using topological features could not achieve a higher performance than that of conventional features for every score in the nested fivefold cross-validation. However, the predictive model using conventional features exhibited extremely low sensitivity (0.383) and specificity (0.369) in a test. In contrast, topological features could helped address this issue, achieving at least a sensitivity of 0.415 and a specificity of 0.622. This implies that topological features could provide additional information for the conventional model, whereas conventional features could also be informative. In addition, the predictive model using all-combined features could achieve the best scores in one of the nested fivefold CV as shown in Table 4. In particular, the sensitivity had a notable score of 0.908. Then, the best predictive model was constructed using RF with 22 all-combined features selected by LASSO-LR. We could achieve the best predictive model by optimizing multiple parameters, including the number of features, feature selection methods, and predictive models.

Previous studies compared with this study were all performed for the purpose of predicting SVD and non-SVD patients using an independent test dataset (Table 4). The predictive model of our study achieved a higher AUC than conventional features. In addition, our study reported a higher sensitivity than those reported by Xie et al., Tang et al., Xiao et al., and Chieregato et al. Consequently, this study could show high performance, particularly in terms of sensitivity, compared to previous studies using radiomic features or deep learning.

As shown in Supplementary Table 4 (Online Resource 6), the top three features used in constructing the best predictive model in both the validation and test were WD_Ori_GLRLM_RP, b0_ks9_ps2_LL_Hist_Energy, and b0_ks5_ps3_Ori_GLRLM_GLV. WD_Ori_GLRLM_RP represents the run percentage (RP), which indicates the ratio of the actual number of runs to the theoretical maximum number of runs derived from the lung image. This reflected the homogeneity of consolidation of SVD patients with COVID-19 pneumonia, leading to lower values in SVD patients compared to non-SVD patients. The LL filter of b0_ks9_ps2_LL_Hist_Energy could generate low- and low-frequency images, providing more information about the center and/or heterogeneity within COVID-19 pneumonia compared to gray-scale images. SVD patients were more widely distributed than non-SVD patients in the violin plots (Fig. 7). This means that the lesion center of the accumulated b0 map, which indicates the connected components of COVID-19 pneumonia, lost its homogeneity and developed complex structures. b0_ks5_ps3_Ori_GLRLM_GLV represents the gray-level variance showing the distribution of the number of zones relative to the gray level on GLRLM on the accumulated b0 map (kernel size = 5, shifting pixel = 3). The connected components (b0) derived from the consolidation of SVD patients resulted in a low variance of b0_ks5_ps3_Ori_GLRLM_GLV, which showed statistically significant differences in both the validation and test. The statistical tests were performed for both the validation and test, and the p values were lower than 0.05 in both cases. The stability and reproducibility of the distribution of the selected features could be demonstrated by statistically significant differences observed between the two datasets (e.g., validation and test) [47].

In addition, case image plots in feature space were generated to visualize the predictive model in a 2D space (Fig. 8). SVD patients were distributed in the center, while non-SVD patients were distributed outside for the validation. The case image plot in feature space for the test was also distributed in the same manner, although the number of cases was small. The distribution of features could be visualized in case image plot in feature space.

This study has four limitations that should be considered in future studies. First, BN maps and accumulated BN maps were constructed in a two-dimensional space. BN maps in a three-dimensional space should be analyzed, since pneumonia spreads throughout the lungs in future studies. Second, the database used in this study was released in August 2020 and mutated coronavirus variants such as Delta or Omicron have emerged worldwide. In addition, long COVID periods were not considered in this study. Therefore, more various patterns of COVID should be tested. Third, clinical features were not considered in the predictive model. Ning et al. constructed a hybrid model combining clinical and CT image features, which improved the AUC [18]. A hybrid model should be constructed in future studies. Fourth, external tests were not conducted for model validation. In this study, a predictive model of SVD was constructed using a dataset from two institutions. Due to the imbalance in the number of cases between the institutions, validation and test datasets were selected from both institutions. Therefore, external tests should be conducted in the future.

## Conclusion

This study demonstrated the construction of a predictive model of SVD in patients with COVID-19 pneumonia using topological properties. Accumulated BN maps could visualize topological features of COVID-19 pneumonia and a predictive model using both topological and conventional features could achieve an AUC of 0.854 and a sensitivity of 0.908 in a test fold. These results suggested that topological image features could characterize COVID-19 pneumonia at an early stage as SVD.

## Supplementary Information

Below is the link to the electronic supplementary material.

Supplementary file1 (PDF 73 KB)

Supplementary file2 (PDF 263 KB)

Supplementary file3 (PDF 55 KB)

Supplementary file4 (XLSX 29 KB)

Supplementary file5 (XLSX 15 KB)

Supplementary file6 (PDF 76 KB)

Supplementary file7 (PDF 48 KB)

## Data Availability

The iCTCF database. https://ngdc.cncb.ac.cn/ictcf/Resource.php
