# Supplementary figures and images for: Predictive models of severe disease in patients with COVID-19 pneumonia at an early stage on CT images using topological properties
Source: Radiol Phys Technol. 2025 Apr 28;18(2):534–46. doi: 10.1007/s12194-025-00906-1 (PMC12103364; doi:10.1007/s12194-025-00906-1)

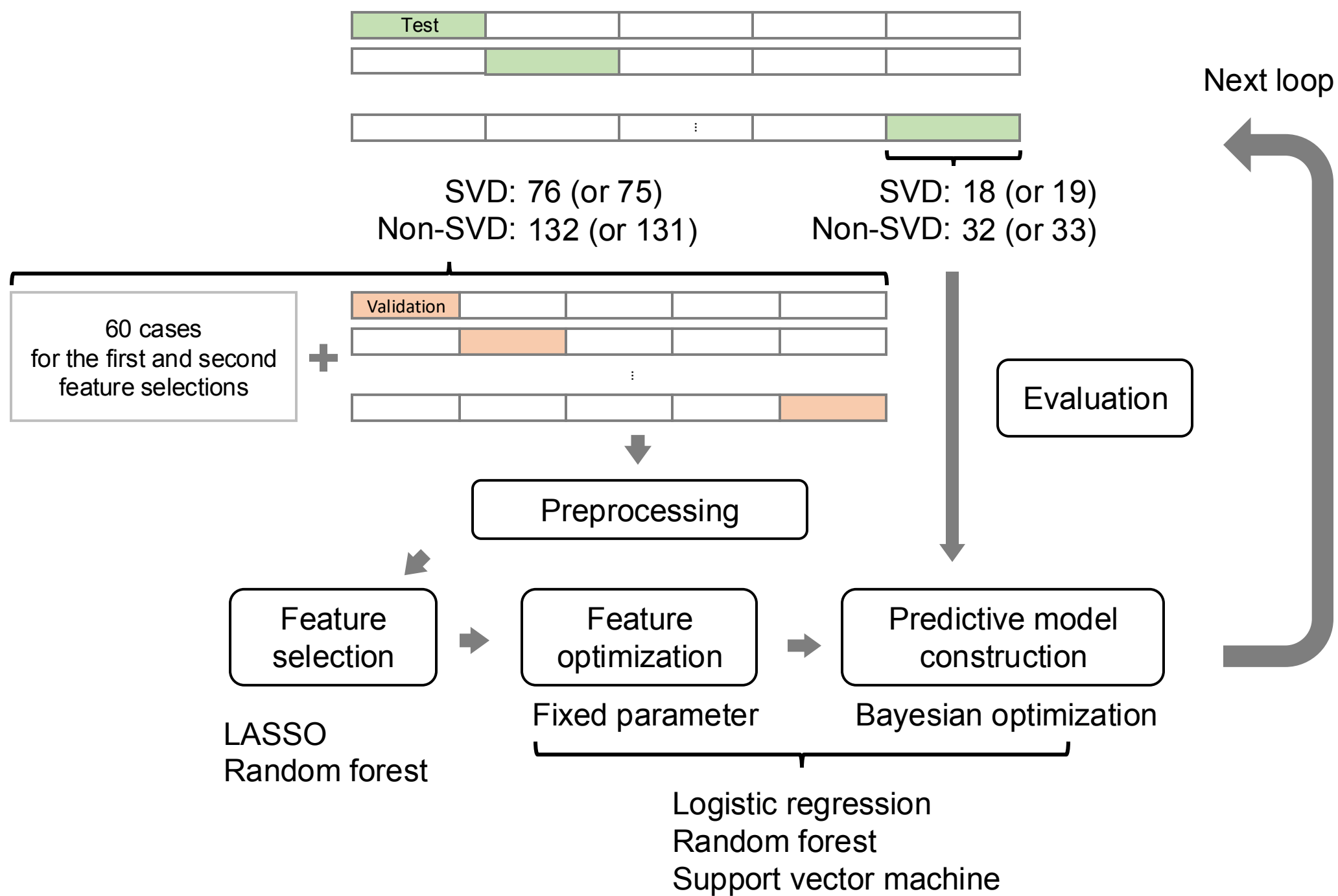

Supplement: Supplementary file 1 — Supplementary file1 (PDF 73 KB) [file 12194_2025_906_MOESM1_ESM.pdf]
