# Supplementary material for: Predictive models of severe disease in patients with COVID-19 pneumonia at an early stage on CT images using topological properties
Source: Radiol Phys Technol. 2025 Apr 28;18(2):534–46. doi: 10.1007/s12194-025-00906-1 (PMC12103364; doi:10.1007/s12194-025-00906-1)
Supplement: Supplementary file 2 — Supplementary file2 (PDF 263 KB) [file 12194_2025_906_MOESM2_ESM.pdf]

Method for determining the initial threshold value for accumulated BN maps

The initial threshold value for accumulated BN maps was determined using the Dice's similarity coefficient (DSC) between binary image and pneumonia region (Supplementary Figure 2 (a)). An array of binary images was created by multiple-threshold values from 0 to 255 with increments of 1 (Supplementary Figure 2 (b)). The DSCs between each binary image and the pneumonia regions was calculated. Then, the relationships between threshold values and the mean DSC of the 60 patients were plotted as shown in Supplementary Figure 3. As a result, the DSC curves initially remained stable, then gradually increased and decreased (Supplementary Figure 3). The onset of DSC change indicates the beginning of binary processing in the pneumonia region. A threshold value of 50 was considered appropriate as a margin for the onset of DSC change. Therefore, the initial threshold value for the accumulated BN maps was determined to be 50 based on an approximate estimate.

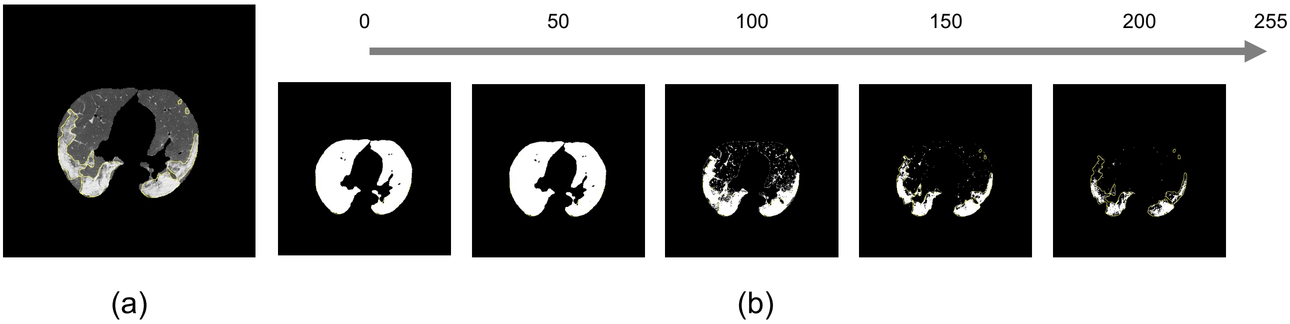

Supplementary Fig. 2 (a) Pneumonia region overlapping lung image (b) Changes of the binary images by multiple-threshold values

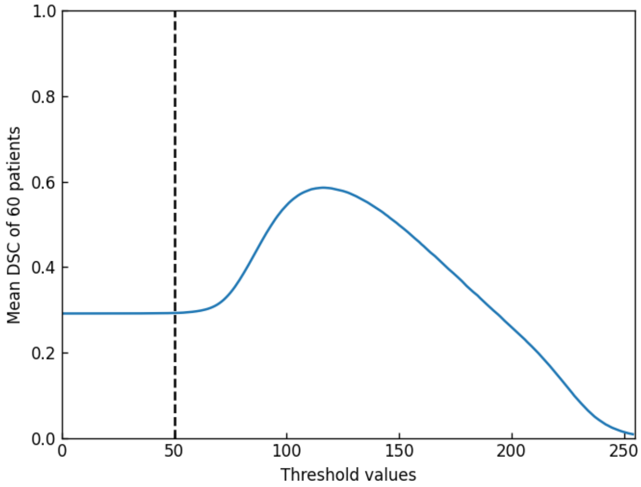

Supplementary Fig. 3 Relationships between threshold values and the mean DSC of the 60 patients
