# Supplementary material for: Predictive models of severe disease in patients with COVID-19 pneumonia at an early stage on CT images using topological properties
Source: Radiol Phys Technol. 2025 Apr 28;18(2):534–46. doi: 10.1007/s12194-025-00906-1 (PMC12103364; doi:10.1007/s12194-025-00906-1)
Supplement: Supplementary file 3 — Supplementary file3 (PDF 55 KB) [file 12194_2025_906_MOESM3_ESM.pdf]

Supplementary Table 1 Ranges of the hyper parameters for Bayesian optimization

Algorithm	Hyper-parameter	Range of the grid search
Logistic regression	penalty	[l2, elastic net]
	C (regularization term)	[0.01, 100]
	ratio	[0.00001, 1]
Support vector machine	kernel	[linear, rbf, sigmoid, polynomial]
	C (regularization term)	[0.00001, 1000]
	gamma (kernel coefficient)	[0.00001, 10]
Random forest	max_depth	[10, 10000]
	Number of estimators	[10, 100]
	Min_sample_split	[2, 5]
	Min_sample_leaf	[2, 5]
	criterion	Gini, entropy
