# Supplementary material for: Predictive models of severe disease in patients with COVID-19 pneumonia at an early stage on CT images using topological properties
Source: Radiol Phys Technol. 2025 Apr 28;18(2):534–46. doi: 10.1007/s12194-025-00906-1 (PMC12103364; doi:10.1007/s12194-025-00906-1)
Supplement: Supplementary file 6 — Supplementary file6 (PDF 76 KB) [file 12194_2025_906_MOESM6_ESM.pdf]

Supplementary Table 4 Selected features of all-combined features for the best predictive model

Feature names	LASSO coefficients	p-values	
		Validation	Test
WD_Ori_GLRLM_RP	-1.76	<0.01	<0.01
b1_ks9_ps3_Ori_GLCM_Entropy	-1.34	<0.01	n.s.
WD_HL_Hist_Entropy	-1.33	n.s.	<0.05
b0_ks9_ps2_LL_Hist_Energy	0.999	<0.01	<0.05
b0_ks5_ps3_Ori_GLRLM_GLV	-0.951	<0.01	<0.05
WD_LH_GLCM_AutoCorrelation	0.875	n.s.	n.s.
WD_HH_GLCM_AutoCorrelation	0.858	n.s.	n.s.
WD_LH_GLCM_Dissimilarity	-0.827	n.s.	n.s.
WD_HL_GLCM_Correlation	0.802	<0.05	n.s.
WD_LH_Hist_Kurtosis	-0.768	n.s.	n.s.
b0_ks9_ps2_LL_NGTDM_Busyness	-0.682	<0.01	n.s.
b0_ks11_ps3_HL_GLSZM_GLN	0.681	<0.05	n.s.
b1_ks11_ps2_Ori_Hist_Min	-0.62	<0.01	<0.01
WD_Ori_GLSZM_SZLGE	-0.606	n.s.	n.s.
b1_ks5_ps4_HH_Hist_Entropy	0.592	<0.01	n.s.
b1_ks11_ps3_Ori_GLSZM_ZSV	0.555	<0.05	n.s.
WD_Ori_GLSZM_LZE	-0.527	<0.01	<0.01
WD_LH_NGTDM_Strength	-0.526	n.s.	n.s.
b0_ks7_ps2_LL_Hist_Energy	-0.518	<0.01	n.s.
b1_ks11_ps4_HL_Hist_Entropy	-0.509	<0.01	n.s.
WD_Ori_GLCM_Correlation	0.504	<0.01	<0.01
b1_ks5_ps2_LL_NGTDM_Complexity	0.497	<0.05	<0.05

LASSO: Logistic regression with least absolute shrinkage and selection operator; n.s.: not significant
