# Supplementary material for: Predictive models of severe disease in patients with COVID-19 pneumonia at an early stage on CT images using topological properties
Source: Radiol Phys Technol. 2025 Apr 28;18(2):534–46. doi: 10.1007/s12194-025-00906-1 (PMC12103364; doi:10.1007/s12194-025-00906-1)
Supplement: Supplementary file 7 — Supplementary file7 (PDF 48 KB) [file 12194_2025_906_MOESM7_ESM.pdf]

Supplementary Table 5 Accuracies of the quadratic discriminant analysis (QDA) with combinations of the three features

Feature 1	Feature 2	Validation	Test
Ori_GLRLM_RP	b0_ks9_ps2_LL_Hist_Energy	0.662	0.641
Ori_GLRLM_RP	b0_ks5_ps3_Ori_GLRLM_GLV	0.689	0.744
b0_ks9_ps2_LL_Hist_Energy	b0_ks5_ps3_Ori_GLRLM_GLV	0.671	0.667
